# National support to public health research: a survey of European ministries

**DOI:** 10.1186/1471-2458-9-203

**Published:** 2009-06-25

**Authors:** Cláudia Conceição, Alexandra Leandro, Mark McCarthy

**Affiliations:** 1 Escola Nacional de Saúde Pública, Universidade Nova de Lisboa, Avenida Padre Cruz, 1600-560 Lisboa, Portugal; 2 UCL Department of Epidemiology and Public Health, University College London, 1-19 Torrington Place, London WC1E 6BT, UK

## Abstract

**Background:**

Within *SPHERE *(Strengthening Public Health Research in Europe), a collaborative study funded by the European Commission, we have assessed the support for public health research at ministry level in European countries.

**Methods:**

We surveyed the health and science ministries in 25 EU countries and 3 EEA countries, using a broad definition of public-health research at population level. We made over 600 phone calls and emails to identify respondents and to gain answers. We gained formal replies from 42 out of 56 ministries (73% response) in 25 countries. There were 22 completed questionnaires (from 25 ministries), 6 short answers and 11 contacts declaring that their ministries were not responsible for public health research, while in 14 ministries (both ministries in three countries) no suitable ministry contact could be found.

**Results:**

In most European countries, ministries of health, or their devolved agencies, were regarded as the leading organizations. Most ministries were able to specify thematic areas for public-health research (from three to thirty), and others ministries referred to policy documents, health plans or public-health plans to define research priorities. Ministries and their agencies led on decisions for financial support of public-health research, with less involvement of other external organisations compared with the process of identifying priorities. However, the actual funds available for public health were not easily identifiable. Most ministries relied on general academic means for dissemination of results of public-health research, while ministries get information on the use of public-health research usually through informal means. Ministries made suggestions for strengthening public-health research through initiatives of their own countries and of the European Union: as well as more resources, improving coordination was most frequently suggested.

**Conclusion:**

There is no common approach to support for public-health research across Europe, and significant gaps in organisation and funding. Health ministries and national agencies value exchange between researchers and policy-makers, civil society organizations, and academic and public authorities, and the application of public-health research results. There would be benefits from better processes of priority setting and improved coordination for research, at regional, national and European levels.

## Background

Health research has wide societal support, because of its collective and altruistic benefit, and because it can particularly help the disadvantaged in society – children, the sick and the poor [1]. It addresses problems of general concern, and has an orientation towards providing solutions. Health research can be divided into three fields: processes at basic biological (molecule, cell level); clinical research (diseases, and interventions on individuals); and public-health research (providing knowledge of the causes and control of disease and promotion of health at population level).

The European Union Sixth Framework Research Programme 2002–2006 substantially increased levels of funding for biomedical research compared with previous years.

But it provided very limited funding for public-health research, and separated it within a composite area of 'policy' research [2,3]. *SPHERE *(Strengthening Public Health Research in Europe), a collaboration of 19 partners in 13 European countries, was developed in response to a call from the European Commission's Directorate for Research, within the policy research strand, to identify priorities in public-health research at the European level, and advise how it can be strengthened and most effectively integrated with European health policy [4].

*SPHERE *had two main objectives: to undertake bibliographic reviews of public health research literature across six fields; and to assess perspectives on public-health research priorities of different stakeholders. Because support for health research in Europe is predominantly a role of national governments, an important element of the work was to map and assess the contributions of ministries of science and ministries of health. The results of the surveys of these national ministries are reported here.

## Methods

### Defining public-health research

Practice of public health varies across countries [2,5], and the meaning of 'public health research' varies also [6]. The word 'public' in English indicates a collective rather than individualist approach, including governmental and non-governmental associations, and 'health' is used to indicate both the prevention and treatment of disease: the usual measures of outcome are incidence of disease, morbidity and death, rather than states of 'wellbeing'.

Public-health research operates at a complex level between scientists, individuals and society. And the issues that public-health research addresses – how to improve the health of the population, and how to ensure the effective and efficient organisation of health care – are of direct concern to governments themselves. For *SPHERE*, the following definition of public health research [7], which gave a broad meaning, was used:

"Public-health research refers to the organized quest for new knowledge to protect, promote and improve people's health. It:

• is undertaken at population or health services level, in contrast to laboratory (cellular) or clinical (individual) health research;

• differs from public-health practice (which also uses scientific methods), as it is designed to obtain generalisable knowledge rather than to address specific programmes for service delivery;

• is usually goal-oriented, addressing questions of policy relevance, and may be published in either academic journals or reports; and

• uses a range of observational methods, including surveys, registers, data sets, case studies and statistical modelling, and draws on disciplines including epidemiology, sociology, psychology and economics, and interdisciplinary fields of environmental health, health promotion, disease prevention, health-care management, health-services research and health-systems research."

### Instruments and processes of data and information collection

The main instrument used to gather information was a questionnaire (mainly composed on open ended questions) covering topics as: priorities for public health research; fund allocation; evidence of success in development of knowledge; relationship between researchers and funders; relationship between national health plans and research supported; and suggestions for strengthening of future public health research. We also used web searches in national websites and email interaction with respondents. The questionnaire is provided as Additional file 1. The questionnaire included the working definition of public health research stated earlier.

### Ethical Approval

In the European Commission peer review process, it was agreed that *SPHERE *did not require ethical approval.

### Study population

We undertook the study in twenty-eight countries, in 2006, of the European Union and European Free Trade Area. The National School of Public Health, Lisbon, Portugal, initially surveyed the 15 pre-2005 EU countries, and Iceland, Norway and Switzerland, and Kaunas Public Health Centre, Lithuania, surveyed the 10 accession countries joining the EU in 2005.

Recognising the considerable difficulty in gaining information by questionnaire, especially one in English sent to government departments, a two-phase process was designed, lasting over a period of 16 months. There were four pathways to identify contacts on the 56 ministries/departments (Additional file 2): ministry websites to identify International Liaison departments; contacts forwarded to us from within Ministries; delegates of the European Medical Research Councils; and contacts with *SPHERE *partners to get help to reach their ministries.

In Phase 1, the questionnaire was sent by email (from Lisbon, ENSP) and by post (from Lithuania, Kaunas Public Health Centre) at the beginning of 2006 (for Norway and Switzerland, in May 2006, and Iceland in February 2007) and continuing until the end of October. For the 15 EU countries, 411 emails or letters were sent – approx 60% gaining replies – and 209 phone calls. The range of contacts per country was from a minimum of 6 to a maximum of 69, representing an average workload of 23 contacts per country and 11 per ministry.

In Phase 2, led by the National School of Public Health, Lisbon, Portugal, a synthesis report (the 'Preliminary Assessment'), which included findings from all parts of *SPHERE*, was sent at the end of February 2007, with a deadline for responses of mid- April. In this phase, nine phone calls were made, 155 emails were sent and 91 returned. Ministries that had already answered the questionnaire were asked for comments and corrections, while the other ministries were asked both for comments and to complete the re-sent questionnaire. 18 ministries provided answers: 13 provided comments (usually about their own country), and 5 gave no comments or amendments, and one new ministry completed the questionnaire.

Finally there were answers from 42 ministries: 22 completed questionnaires from 25 ministries (3 countries organized themselves providing a single answer), 6 short answers (information sent by ministries but not as fully-filled questionnaires), and 11 ministries saying they did not support public health research. In 14 ministries (both ministries in three countries) no suitable ministry contact could be found (Table 1).

**Table 1:** Responses by country and ministry.

**Country**	**Ministry of Health**	**Ministry of Science**	**Country**	**Ministry of Health**	**Ministry of Science**
Austria	+	×	Latvia	×	-
Belgium	+	+	Lithuania	/	/
Cyprus	-	×	Luxembourg	-	-
Czech Republic	/	×	Malta	+	+
Denmark	+	+	The Netherlands	+	+
Estonia	+	×	Norway	+	×
Finland	+	/	Poland	-	-
France	+	+	Portugal	+	+
Germany	×	+	Slovakia	+	-
Greece	-	×	Slovenia	-	×
Hungary	+	-	Spain	+	×
Iceland	-	-	Sweden	+	+
Ireland	-	×	Switzerland	+	+
Italy	+	-	United Kingdom	/	/

Key: + full reply / limited reply - no contact × not responsible

## Results

Information was provided for 42 ministries in 25 European countries, but despite our best efforts over 18 months, it was not possible to find key contacts in 14 ministries. Not all components of the questionnaires were answered: the responses therefore have varying denominators, as indicated in the results. The analysis relies heavily on the information provided, and it is not possible to validate the replies: however, there were few criticisms of the preliminary report provided to the ministries for their comment. The research also showed that, in some countries, areas such as environmental or occupational health research are supported by other ministries that we did not contact, and are therefore not fully reflected in the results.

Drawing on the multiple sources of information (questionnaire, websites and email contacts), the leading ministry for public health research in 19 countries is the Ministry of Health, and in 4 countries the Ministry of Science, with one country (France) drawing equally from both ministries. The questionnaires covered three broad areas: priorities; funding; and implementation.

### Priorities

The ministries saw themselves, or their direct agencies (e.g. boards or councils), as the leading organizations for determining public-health research priorities (Table 2). Only three ministries responded that they had "no special structure".

**Table 2:** Type of advisory organizations that are involved in the process of identification of PHR priorities

**Type of advisory organizations**	Number of ministries
Autonomous public structures/agencies	18
Ministries	13
National and regional levels coordinating structures	5
Non governmental structures/charities/private	4
No special structure	3

Number of answers: 23 ministries

Ministries used simultaneously different processes of public-health research priorities identification (Table 3). Several ministries described a "bottom-up" process: "Research groups can choose what they want to propose for funding based on their own "feeling" of what is more important, timely or just 'trendy' (therefore more likely to be funded)". More commonly, there was a linear process from ministry to researchers: for example, "In the Ministry of Health [is] the National Committee of Research, constituted by several experts in the area, which defines the priorities of the public-research within the objectives of the [national health plan]". Some countries have well-differentiated public organizations/agencies that collaborate with ministries in defining priorities, perhaps leading consultation though "conferences or meetings with patient organizations", or participation "in public debates with universities, public authorities and funding agencies". One country described "Innovation accelerating research platforms", designed to join existing areas of high research potential with business community growth potential. For some countries coordination at the regional and national levels was the most important characteristic of the process.

**Table 3:** Processes of public health research priorities identification

**Processes of public health research priorities identification**	Number of ministries
"Experts"/scientific committees	7
No specific process	6
Coordination between different agencies	5
"Bottom-up" processes	5
Strategic foresights as support to process	5
Consultations with different organizations	4
Coordination between national and regional levels	2

Number of answers: 18 ministries

Most ministries were only able to specify thematic areas for research and others ministries referred to policy documents, health plans or public health plans to define research priorities (although different ministries may regard different documents, in the same country, as setting research policy). For others priorities were related to national research plans or to health research programmes of national agencies. One respondent answered that priorities are "very broad, not specifying any particular field", indicating that there were no explicit priorities (Table 4).

**Table 4:** Types of priorities on PHR pointed by ministries

**Types of priorities on PHR pointed by ministries**	Number of ministries
Thematic areas with no additional information	12
Priorities related to public health policy or health policy (national health plans, public health plans)	8
Priorities related to research programmes of national agencies or themes of calls for research projects	5
Priorities related to national research plans	4

Number of answers: 27 ministries

Research themes (from three to thirty) were described in terms of:

• Areas of research (examples: health-services/health-care research, research on prevention and health promotion, and environmental health);

• Diseases (examples: cancer, cardiovascular diseases, diabetes, respiratory illnesses, and infectious diseases including HIV/AIDS); and

• Determinants of health (examples: food safety and nutrition, illegal drug consumption, social inequalities, immigrants and marginal social groups)

Several ministries described processes for developing a health-research agenda. One country explained that it was trying to coordinate ministries and agree priorities, since the ministry of science "can finance health research with no evaluation or previous consultation with health ministry". In another country, "National Research Programmes (NRP) are selected in a bottom-up process. This means that interested groups can submit ideas for new research programmes to the [ministry]... [The] government (...) periodically decides on the topics as well as the financing of one to three new NRP". In another country, the public agency had "made an investigation among (the associations and federations) of all main actors and stakeholders.(...) This investigation resulted in a discussion paper. (...) [Another document] describes the developments in the field of Health Care and Public Health and the challenges the society is facing. The document is aimed to become the guideline for the knowledge & research agenda of the Ministry."

### Funding

The ministries used multiple criteria to fund public health research (Table 5). Values included "scientific excellence", "relevance to the objectives of the call", "importance of the topic", "burden of disease" and "ministerial priority". For several ministries, the principal criterion was that "the research is intended to provide support to a particular policy decision" or the relationship with needs is stated on the national health plan. Reactive "bottom-up" research responding to the scientific community was present in most countries, while in some research units decide themselves the topics of research: "Major attention is given to the right of the scholars to choose and shape their own problems". In some cases respondents clarified that "These are the general criteria for funding health research and not only for funding public health research". Five ministries said there were no detailed criteria for funding public-health research: "There is a specific call under the subject epidemiology/public health. However, there is no further research topic specification" or " [the ministry] has not established specific criteria to allocate funds for public-health research".

**Table 5:** Ministries' criteria for public health research funds allocation

**Ministries' criteria for public health research funds allocation**	Number of ministries
Characteristics of the financed projects	11
According to political strategies/to support policies/according to national health plan	9
On demand/scholars criteria	6
No specific criteria	5

Number of answers: 23 ministries

Ministries directly (15 out of 19 ministries) and their agencies (11 out of 19 ministries) led on the decisions for financial support of public-health research, with less involvement of other external organisations compared with the process of identifying priorities. In one country, "The decision is made by the Ministry alone. But before the Ministry makes any decision a qualified consultation system with advisory boards is implemented, in order to achieve transparency, high quality and impartiality within the scientific community. In some fields of public-health research, health insurance funds, [country] pension insurance and other organizations, which carry out prevention programs, are involved in the funding decisions. This is due to "the necessity to be able to transfer the results into the practical work more easily".

Processes for allocating funds varied (Table 6). In some ministries, calls are made for specific problems/areas, previously defined or existing within strategic foresights, plans or research agendas. Others acknowledged a dominance of "on demand" research: "there is no specific process", "fund allocation will be done on application" and so "the best programmes are funded". Usually, public agencies, medical councils, panels of independent referees will be involved in the evaluation of the proposals that are eventually financed. However, public agencies, universities and research centres more often received pluriennial funding, while ministries (all responding, 18) funded research for only one to three years.

**Table 6:** Ministries' public health research fund allocation processes

**Processes for funds allocation**	Number of ministries
Related to a research agenda/National Research Plan	10
No research agenda (on demand/investigator led research)	7
Sporadic call for a certain investigation in need	5
In process of definition of a research agenda	3
Through current financing of public institutions that do research (ex public universities; public agencies)	3
Through support research centres with pluriannual ministry's funding	2

Number of answers: 21 ministries

The actual funds available for public health were not easily identifiable. Only two countries could state the allocation to public-health research within their Ministry's annual budget (indicated as 1–2% and 30% respectively). However, it was possible to find more detailed information on money allocated to health research for two other countries [8-10]. In some countries, research is linked to general policy aims, and operational goals, for example in health promotion areas. Other respondents stated that the ministry's budgetary organization did not specify any funding explicitly for public health research, or that it was not separately identified within budgets for broader health research.

**Figure 1:**
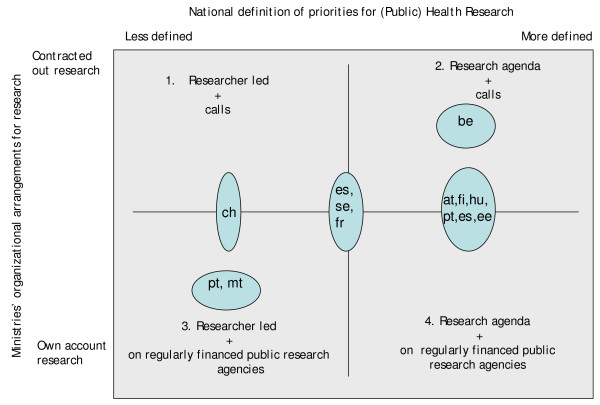
**Countries' contracted out and internal research**. Note: at – Austria; be – Belgium; ch – Switzerland; ee – Estonia; es – Spain; fi – Finland; fr – France; hu – Hungary; mt – Malta; pt – Portugal; se – Sweden

Ministries were asked to describe the balance between "internal" and "contracted out" public-health research. Internal public-health research is considered to be regularly funded, and performed in structures legally dependent on a ministry. Contracted out public-health research may be performed by non-governmental agencies, including private, by applying and through calls for funds available for research projects (mostly additional to regular funding of research agencies and universities). In both settings, however, it may be either researcher-led, or in response to identified research agendas (e.g. national research health plans, pro-active attitude from ministry). Drawing on responses provided by at least one ministry, the predominant position of public health research of eleven countries within four quadrants is internal, with both ministries and researchers developing the agenda (Figure 1).

Countries were asked if the criteria for financing public-health research were explicitly linked to a national health plan. While 11 ministries (out of 17 ministries) stated that it was (and a further 5 said they had no national health plan), this was sometimes qualified: "Yes. A small part of the total funding relates to national health-plans"; "Yes. Very loosely". We also used the internet to examine national health-plans written in English of seven countries: of these, only three mentioned public-health research. Drawing these sources together, finance for public-health research was generally not explicitly linked to national health-plans, and thus to priorities/needs in action.

### Implementation

Eleven ministries (out of 15) described peer review processes to assess the success of public health research. One ministry, however, was currently without an assessment process, and in another ministry the process was administrative rather than peer reviewed. One country had "commissioned an international evaluation" of national public health research overall [11].

The support described for communication/dissemination of results of public health research is presented in Table 7. Strategies were described as "the Ministry stimulates the dissemination of the research results", "we demand that the results are made public" and "there is no specific instrument" and so most ministries relied on general academic means for dissemination (publications on paper and websites, and presentations at scientific meetings and conferences). In some cases research agencies allocated funds specifically for communication strategies, and also held databases of their publications and projects. For one ministry, "a more concise form of the results is considered as an alternative for the detailed versions of the research findings".

**Table 7:** Ministries support to communication/dissemination of results of PHR

**Level of support for communication**	Number of ministries
Poorly defined strategies (publications – paper and website, participation on workshops and conferences, scientific meeting attendance and result publishing eligible for funding)	11
Communication strategy in place	4
Projects budgets allocate specifically for communications strategies	4

Number of answers: 15 ministries

Ministries get information on the use of public-health research findings in the field usually only by informal means: "there is no such formal follow-up action", "there is no systematic feed-back". Ministries have "regular meetings of public health practitioners", and learn by "going to conferences, participating in activities of other ministries or institutions" and publications. One ministry explained that, because the public-health services are provided by municipal authorities, "the Ministry does not know whether research findings are applied by health practitioners or not". In the same country, a national evaluation found that "only 9% of the policy memorandums contain provisions for evaluating achieved results. And only half of the municipal health-departments carry out a systematic evaluation of their programmes" (Table 8).

**Table 8:** Ministries' processes of getting information on the application of public health research findings in the field, by public health practitioners

**Processes of getting information on uses of public health research**	Number of ministries
Informal ways (Websites, meetings with public health practitioners and researchers, papers, consultations)	7
No formal process of getting information on application of new knowledge	2
Evaluation processes	2

Number of answers: 11 ministries

Although 28 ministries provided descriptions of agencies supporting public-health research, only 14 replies were given about processes of coordination of public health research (Table 9). Most replies were negative: "no direct coordination takes place. Different interest groups (e.g. health-care committees and patient organizations) however, are actively following the area and are occasionally in contact with policy makers"; "there is no coordination". However, one national agency on health research conducted a study to describe the developments expected in health and the research required, which then formed the basis of a policy paper.

**Table 9:** Processes of the ministries initiative of coordination (between needs identified by users, scientific community and policy makers and resources for research) of public health research

**Processes of public health research coordination**	Number of ministries
Not existing/no direct coordination	8
Interministerial working groups/memberships or advisory boards of research agencies	6
To prepare and use coordinating instruments: discussion papers, strategic foresights, plan, budget, guidelines for research agenda	4
Financed projects steering committees (follow up, evaluation)	2
Informal contacts between policy makers and interest groups	2

Number of answers: 14 ministries

There was said to be a national board of health research in 17 of 20 ministries which gave replies. Drawing from the replies given, a national board of research is generally understood as a set of governmental bodies (in Switzerland, it is a private body with a federal mandate) with functions to advise government and ministries on the area of research policy, fund research, coordinate research funding of their ministry, coordinate researchers (public or private organizations, research teams and individuals) and coordinate wider research funding streams (public and private). However, when asked if they had a national board of public-health research as well, only 3 ministries said "Yes", although there were sometimes boards on "Biology and Medicine" or "Health Sciences" that included "epidemiology/public health" inside national coordinating boards of research.

It is possible to identify different levels in the management of scientific knowledge production, dissemination and implementation. Some of the national agencies intervene in the identification of priorities and are more or less concentrated in the public availability of scientific knowledge, in particular the research commissioned to and/or funded by them: examples include ZonMW (Netherlands Organization for Health Research), INSERM (French National Institute of Health and Health Research), FAS (Swedish Council for Working Life and Social Research) and SNSF (Swiss National Science Foundation). Others are centres of expertise: RIVM (the Netherlands National Institute for the Public Health and Environment) and NICE (English National Institute for Health and Clinical Excellence). We found that organisations are investing in communication platforms and tools, like databases and newsletters, designed to make the results of the funded research public. Additionally, the ongoing development of implementation workshops and networks designed to promote the transfer of guidance and innovation into the health system, considering health-care professionals, researchers and policymakers.

Finally, ministries were invited to give suggestions on how to strengthen public-health research at national level through initiatives of their own countries and of the European Union (Tables 10 and 11). Several suggestions were given by each ministry. As well as more resources, improving coordination was the most frequent suggestion: "strengthen coordination between different policy makers competent in the area of public health, universities and other research institutions", "establish a permanent forum involving researchers, funding agencies and users (health professionals, policy makers, general public)", "create support for closer collaboration between researchers in medicine, social sciences and humanities". More attention to research priorities was required, for example: a "clear overall process for defining priorities", to "develop priority-setting 14 methodologies" and to "set up R&D priorities to meet needs of new scientific knowledge". Evaluation, access to information, dissemination and knowledge transfer were also recommended.

**Table 10:** Ministry suggestions for strengthening public health research at national level

**Suggestions for strengthening public health research at National level**	Number of ministries
Increase allocated resources (money, human resources, training, infrastructure)	11
Improve coordination (policy makers, health professionals, general public, universities, other research institutions, researchers, research funders)	8
Make explicit the criteria for choosing priorities	4
Structure processes of research evaluation	4
Increase the number of public health research topics in national health programmes	3
Increase communication of research findings and knowledge transfer	3

Number of answers: 15 ministries

**Table 11:** Ministry suggestions for strengthening public health research at European level

**Suggestions for strengthening public health research at European level**	Number of ministries
Increase international collaboration and networking	13
Increase European focus on the area of public health research	8
Increase and improve the quality of European funding	5
Better access to information	1

Number of answers: 17 ministries

At European level, the most frequent call was to "support collaborative research and networking" and to increase EU focus on the area of public-health research. There was a call for "reinforcing cross-activities between SANCO [EU Directorate for Health] programmes and the thematic priority of Health in the [EU Directorate for Research] 7th Framework Programme". Better funding was also recommended for infrastructures: "human capital training and mobility", "training and exchange of students/scholars and practitioners", "renewal of infrastructure, train scientists, promote interregional cooperation". One ministry stated that "the Framework Programme for Research and the Programme of Community Action in the field of Public Health are not simply an additional source of money but are very valuable in stimulating transnational networking and joint projects with truly European added value". Some ministries went further and suggested that "international collaboration within the ERA-NETs is one option" and "ERA-NETs ... including research on public health as a full integrated part". It was also suggested that the EU could "approach local policy makers, ministerial authorities, and research councils of the Ministry of Health" – suggesting that at present their contact is limited (17 countries). [ERA-NETs are mechanisms sponsored by the EU Directorate for Research in coordinating nation research organisations at European level.]

## Discussion

This study is the first to describe governmental support to public-health research in Europe. We used a structured open-ended questionnaire for data collection. We engaged with the health and science ministries in 28 European countries through an iterative process, following-up the questionnaires initially sent using email and phone calls, by sending preliminary results to respondents for their review (Phase II of this project), and integrating the comments. The project itself, developed by researchers, aimed to build relationships and work with policy-makers.

Coordination and dialogue between the national ministries of health and science was weak. Despite providing the information that we were contacting both ministries, and asking for help when we were having difficulties in finding key contacts, only three countries provided shared answers. The responses to the question on priorities for research illustrates this difficulty, as different priorities, or documents for reference, might be provided by the two ministries in the same country. The report also reflects the difficulties of agreeing (in English, not in a language of the country) on the concept of public health, and the boundaries between public health and public health research. Moreover, the distinction between health priorities and health research priorities was not clear when speaking about priority-setting, or questions on how national health plans were related to criteria of funding research.

This study found that, for priority-setting in public-health research, ministries pointed to different degrees of interaction and participation between researchers, funders and society. Generally, the processes were poorly defined and not specific to public health research. There was a notable lack of information on the funding allocated to public health research. Making the different components explicit, as well as their correlation, was sometimes seen to be impossible. A few respondents reflected over the complexity of managing public health research and/or exposed the fragilities of the system.

Priority setting is an evolving process, starting with what is possible on a national context at each point in time. The Council on Health Research for Development [1] proposes that the different steps and important issues health research priority setting are: identification of the main resources for health research; inclusiveness (choice of those who should be involved in the process); methods, tools and criteria for priority setting; equity and legitimacy (process that promotes equity and uses fairness and legitimacy); communication, dissemination and feed-back of information; place for "curiosity-driven", "researchers led research"; translating priority health issues into priority health research issues; implementing, monitoring and evaluating; and sustaining priority setting as a function of the national health research system. The questionnaire isolated some of these different steps, as identifying priorities and resource allocation, but most answers did not reflect those different steps, or the interaction between them. Priority-setting is a process that involves budget decisions, monitoring and evaluation: this was not the case for most of the respondents.

Decisions relating to the financial support for public-health research were more centred in the ministries themselves than the processes of public health research priorities identification. Where budget concerns predominated, the consulting process was less extensive.

Some ministries stated that they are considering improving the evaluation processes of research funded projects. One country provided a document with an external evaluation of the national public health research [11]. The authors write: "Public health research generates systematic knowledge about the health of the population, as well as the factors which influence public health and its distribution. Public health research studies and evaluates measures aimed at the preservation and improvement of the health of the population. Studies looking at the significance of societal structure, working life, environment, health behaviours and healthcare systems for population health are in focus". This definition "is based on a broad concept of health. The definition is meant to include monitoring and surveillance of population health as well as health services research". Similar to other work packages from *SPHERE *[12-18], this evaluation concluded that intervention research is currently less well developed than descriptive studies and studies on causes of diseases. It highlights the existence of many small units in countries, which are challenged by the increasing complexity and costs of multidisciplinary public health research.

Many national agencies fulfil the functions of "knowledge translation platforms" [19] which include: goals related to improvement of application of knowledge; updated databases of research programmes, publications, policy documents; promotion of meetings and conferences between researchers and policy makers to discuss policy implications of new knowledge; regular consulting (needs) and communication (of research findings) with patient organizations, universities, public authorities and research funders. The data gathered in this study show that ministries often do not know if research findings, or best known practices, are being implemented. One ministry gave information on an evaluation of the last 10 years of public health services and concluded that there was no systematic evaluation of programmes, and that indeed very few programmes undergo evaluation.

## Conclusion

Ministries of health take the lead for public-health research in most European countries, but there is no common approach. There are significant gaps in the organisation and funding of public-health research, and better processes are needed for priority-setting, and the accumulation, dissemination and implementation of scientific knowledge.

A European strategy for public-health research should be based on equity and accountability, be sufficiently stable to consider middle and long-duration policy and scientific approaches, and flexible enough to integrate new concerns, stakeholders and methodologies. The technical and political control and instruments for management of public-health research requires improved coordination between researchers, funding agencies and society, both at national and European levels.

## Competing interests

The authors declare that they have no competing interests.

## Authors' contributions

The study was conceived by MM, designed by CC, undertaken by CC and AL, and writing was by CC, AL and MM. All the authors read and approved the final manuscript.

## Pre-publication history

The pre-publication history for this paper can be accessed here:



## Supplementary Material

Additional file 1 **Questionnaire**. Questionnaire used for the study, including cover letter. Click here for file

Additional file 2 **National ministries of health and ministries of science**. List of ministries contacted Click here for file
