# Supplementary material for: National support to public health research: a survey of European ministries
Source: BMC Public Health. 2009 Jun 25;9:203. doi: 10.1186/1471-2458-9-203 (PMC2713230; doi:10.1186/1471-2458-9-203)
Supplement: Additional file 2 — National ministries of health and ministries of science. List of ministries contacted [file 1471-2458-9-203-S2.doc]

**National ministries of health and ministries of science**

**Austria**: Federal Ministry for Health and Women; Federal Ministry for Education, Science and Culture

**Belgium**: Federal Public Service Health, Food Chain Safety and Environment; Belgian Federal Science Policy Office

**Cyprus** : Ministry of Health; Ministry of Education and Culture

**Czech Republic**: Ministry of Health; Ministry of Education, Youth and Sport

**Denmark**: Ministry of the Interior and Health; Ministry of Science, Technology and Innovation

**Estonia**: Ministry of Social Affairs; Ministry of Education and Research

**Finland**: Ministry of Social Affairs and Health; Science and Technology Policy Council of Finland

**France**: Ministry of Health and Solidarity; Ministry of Education, Higher Education and Research

**Germany**: Federal Ministry of Health; Federal Ministry of Education and Research

**Greece**: Ministry of Health and Social Solidarity, Ministry of Development; General Secretariat for Research and Technology

**Hungary**: Ministry of Health; Ministry of Education and Culture

**Ireland**: Department of Health and Children; Department of Education and Science

**Italy**: Ministry of Health; Ministry of Higher Education and Research

**Latvia**: Ministry of Health; Ministry of Education and Science

**Lithuania**: Ministry of Health; Ministry of Education and Science

**Luxembourg**: Ministry of Health; Ministry for Culture, Education and Research

**Malta**: Ministry of Health, the Elderly and Community Care; Ministry of Education

**Netherlands**: Ministry of Health, Welfare and Sport; Ministry of Education, Culture and Science

**Norway**: Ministry of Health and Care Services; Ministry of Education and Research

**Poland**: Ministry of Health; Ministry of Education and Science

**Portugal**: Ministry of Health; Ministry of Science, Technology and Higher Education

**Slovakia**: Ministry of Health; Ministry of Education

**Slovenia**: Ministry of Health; Ministry of Higher Education, Science and Technology

**Spain**: Ministry of Health and Consumption; Ministry of Education and Science

**Sweden**: Ministry of Health and Social Affairs; Ministry of Education, Research and Culture

**Switzerland**: Federal Office of Public Health; State Secretariat for Education and Research

**United Kingdom**: Department of Health; Department of Trade and Industry; Office of Science and Technology
