# Supplementary material for: National support to public health research: a survey of European ministries
Source: BMC Public Health. 2009 Jun 25;9:203. doi: 10.1186/1471-2458-9-203 (PMC2713230; doi:10.1186/1471-2458-9-203)
Supplement: Additional file 1 — Questionnaire. Questionnaire used for the study, including cover letter. [file 1471-2458-9-203-S1.doc]

**Additional file 1**

**strengthening public health research in europe**

**January 2006**

**Dear Sir/Madam,**

The European Public Health Association (EUPHA), through the Faculty of Public Health, University College London, United Kingdom, is undertaking a project, *SPHERE* (Strengthening Public Health Research in Europe), that will describe public health research at the European level, including support by national governments, and advise how it can be strengthened and most effectively integrated with European health policy.

The study will be undertaken by a consortium of European public health experts from EU Member States. We are asking your collaboration for a part of the study that is undertaken by the National School of Public Health, Lisbon, Portugal. The main objectives are to obtain information from Ministries of Health and of Science and Education about the support for public health research, evidence of success in knowledge development, use in the field and uptake by policy makers. We have designed a questionnaire to fulfil this objective, and are kindly asking your cooperation in completing it.

The coordinators of the project will send the first report to national governments for consultation, and we would be most grateful for your Ministry/Department’s involvement.

Despite consulting with partners and conducting internet searches, we remain unsure about some of the email addresses for the International Department of the Ministry. If there is a department more oriented to international contacts than yourselves, we would be most grateful if you could pass their email address to us.

The SPHERE project encompasses a large range of public health issues, and as such, other public health research and training institutions within Europe will also be approached to participate through the completion of questionnaires related to these topics.

We thank you for your participation in this project.

Yours faithfully,

Mark McCarthy

Professor of Public Health, University College London, United Kingdom

***SPHERE***

Strengthening Public Health Research in Europe

Ministry of…….

Email: …

***Before completing this questionnaire please note the following:***

1 - **Public health research** usually refers to the organized quest for new knowledge to protect, promote and improve people’s health. As public health research can have some different understandings we would like to strength that in this study we are referring public health research:

i) that is undertaken at **population or health services level**, in contrast to laboratory (cellular) or clinical (individual) health research;

ii) that differs from public health practice (which also uses scientific methods), as it is **designed to obtain generalisable knowledge** rather than to address specific programmes for service delivery;

iii) that is usually goal-oriented, addressing questions of policy relevance, and may be published in either academic journals or as reports;

iv) that uses a range of observational methods, including surveys, registers, data sets, case studies and statistical modelling, and draws on disciplines including epidemiology, sociology, psychology and economics, and interdisciplinary fields of environmental health, health promotion, disease prevention, health care management, health services research and health systems research.

2 – Where an official document exists on the topics covered, we would be most appreciative if you could please attach them to the questionnaire, either by email, fax, post or simply by including the web link. We would prefer English versions where possible, however, native language documents will also be accepted.

3 – We are sending the questionnaire as [name of the document].doc and pdf. We suggest you to save the file [name of the document].doc in your own computer as [name of the document]_anwer.doc before filling in the questionnaire.

***Part 1 – Personal particulars of respondent***

| Surname: | | Title: | | Initials: | |
| --- | --- | --- | --- | --- | --- |
| First name: | | | | | |
| Position in the Ministry/Department: | | | | | |
| Telephone and fax numbers: | | Country code: | | Area code: | |
| Tel (1) | Tel (2) | | Fax (1) | | Fax (2) |
| e-mail (1) | | | e-mail (2) | | |
| Other information that may help us to contact you: | | | | | |
| If we need to contact you for clarifications, and you are not available, who else can we contact? | | | | | |
| Surname: | | Title: | | Initials: | |
| First name: | | | | | |
| Telephone and fax numbers: | | Country code: | | Area code: | |
| Tel (1) | Tel (2) | | Fax (1) | | Fax (2) |
| e-mail (1) | | | e-mail (2) | | |
| Other information that may help us to contact him/her: | | | | | |

***Part 2 –Ministry support for public health research***

***2.1 - Identification of priorities and criteria for funds allocation.***

| 2.1.**1** - Please name the advisory structures or other organizations that are involved in the process of identification of **public health research** priorities in your country. | | |
| --- | --- | --- |
|  | | |
| 2.1.**2** - Succinctly describe the process of identification of **public health research** priorities in your country. | | |
|  | | |
| 2.1.**3** - What are the priority areas for **public health research** in your country at present? | | |
|  | | |
| 2.1.**4** - Is there an official document on the subject of priorities in public health research?  *Mark the appropriate answer with an X.* | Yes | No |
| If yes, please email, fax or indicate its web address. | | |

| 2.1.**5** - What criteria does your Ministry/Department use to allocate funds for **public health research**? | | |
| --- | --- | --- |
|  | | |
| 2.1.**6** - Which organisations are involved in deciding which criteria will be used for the allocation of funds for **public health research**? | | |
|  | | |
| 2.1.**7** - Is there an official document on the subject of criteria for fund allocation?  *Mark the appropriate answer with an X.* | Yes | No |
| If yes, please email, fax or indicate its web address. | | |

| 2.1.**8** – Briefly describe the process of fund allocation from your Ministry/Department to **public health research**. | | |
| --- | --- | --- |
|  | | |
| 2.1.**9** – How frequently does fund allocation occur? | | |
|  | | |
| 2.1.**10** - For how long are funds allocated? | | |
|  | | |
| 2.1.**11** - Is there an official document on the subject of process of fund allocation?  *Mark the appropriate answer with an X.* | Yes | No |
| If yes, please email, fax or indicate its web address. | | |

| 2.1.**12** – What kind of **public health research** does your Ministry/Department fund?  *Mark the appropriate option with an X.* | |
| --- | --- |
| 1 – The Ministry/Department funds only internal **public health research** (on its own account) |  |
| 2 – The Ministry/Department contracts out all **public health research** |  |
| 3 – The Ministry/Department funds both internal and external (contracted out) public health research.  Please specify: |  |
| proportion of Ministry/Department budget (2006) for internal **public health research** | … % |
| proportion of Ministry/Department budget (2006) for contracted out **public health research** | … % |

| 2.1.**13** - Are funds allocated to **public health research** clearly identifiable in the Ministry/Department budget for 2006?  *Mark all the appropriate options with an X.* | |
| --- | --- |
| Yes |  |
| If so, please specify the % of total budget for research in your Ministry/Department | …% |
| No |  |
| Funds are available for **public health research**, but mixed in with other funding and hard or impossible to link explicitly with public health research |  |
| Other |  |
| Please, specify | |

***2.2 - Relationship between national health plans and criteria of research financed***

| 2.2.**14** - Are the criteria of **public health research** funding related to the national health plan? *Mark* *the appropriate option with an X.* | |
| --- | --- |
| We don't have a national health plan |  |
| Yes |  |
| Please specify in which way: | |
| If yes, please email, fax or indicate the national health plan web address. | |
| No |  |

***2.3 - Evidence of success in development of knowledge***

| 2.3.**15** - Briefly describe the process of evaluation of **public health research** funded by your Ministry/Department. | | |
| --- | --- | --- |
|  | | |
| 2.3.**16** - Briefly describe how your Ministry/Department supports the communication/dissemination of results of **public health research**. | | |
|  | | |
| 2.3.**17** - Is there an official document on the subject of evaluation of success in development of knowledge on public health?  *Mark the appropriate answer with an X.* | Yes | No |
| If yes, please email, fax or indicate its web address. | | |

***2.4 - Uses of the results of research in the field***

| 2.4.**18** - How does your Ministry/Department get information on the application of **public health research** findings in the field, by public health practitioners? | | | |
| --- | --- | --- | --- |
|  | | | |
| 2.4.**19** - Is there a system tracing the implementation of **public health research** data?  *Mark the appropriate option with an X.* | | | |
| Yes | | |  |
| Please describe | | | |
| No | |  | |
| 2.4.**20** - Is there an official document on the subject of the implementation of research findings in the field?  *Mark the appropriate answer with an X.* | Yes | No | |
| If yes, please email, fax or indicate its web address. | | | |

***2.5 - Uptake of the results of research by policy makers***

| 2.5.**21** - What mechanisms or processes allow your policy makers to uptake the results of **public health research**? (please provide examples) | | |
| --- | --- | --- |
| Processes of the initiative and/or responsibility of your Ministry/ Department.  Other processes: | | |
| 2.5.**22** - Is there an official document on the subject of uptake of research results from policy makers?  *Mark the appropriate answer with an X.* | Yes | No |
| If yes, please email, fax or indicate its web address. | | |

***Part 3 – Relationship between research practitioners and research funders***

| 3.**23** - Please provide the organisational reference for institutions/agencies that fund **public health research** (national or regional, public or private). | | |
| --- | --- | --- |
|  | | |
| 3.**24** - Briefly describe the process of coordination (between needs identified by users, scientific community and policy makers and resources for research) of public health research? | | |
|  | | |
| 3.**25** - Is there an official document on the subject of coordination of public health research?  *Mark the appropriate answer with an X.* | Yes | No |
| If yes, please email, fax or indicate its web address. | | |

| 3.**26** - Is there a national board for research?  *Mark the appropriate answer with an X.* | |
| --- | --- |
| Yes |  |
| Please provide a contact and/or the web address of the board | |
| No |  |
| 3.**27** - Is there a national board for **public health research**?  *Mark the appropriate answer with an X.* | |
| Yes |  |
| Please provide contact of the board | |
| No |  |

***Part 4 – Progress of each country in commenting on, and supporting, the Research Directorate-General 7^th^ Framework Programme.***

4. **28** - Please send or indicate the web address where to find the most recent contribution from your government to the DG Research 7^th^ Framework Programme.

4.**29** - If not, please explain the process and national contribution to the DG Research 7^th^ programme.

***Part 5 –***Strengthening of public health research at national level

| This project will integrate different parts to finally advise how public health research can be strengthened and most effectively integrated with European health policy. | | |
| --- | --- | --- |
| 5. **30** – What could be done, at national level, to develop public health research? | | |
|  | | |
| 5.**31** - Is there an official document on the subject of strengthening public health research at national level?  *Mark the appropriate answer with an X.* | Yes | No |
| If yes, please email, fax or indicate its web address. | | |
| 5. **32** - In what ways could the European Union contribute to develop public health research in your country? | | |
|  | | |
| 5.**33** - Is there an official document on the subject of the contribution of the EU to the development of public health research in your country?  *Mark the appropriate answer with an X.* | Yes | No |
| If yes, please email, fax or indicate its web address. | | |

Thank you very much for answering this questionnaire. Should you have further queries, please contact Claudia Conceição or Patricia Barbosa at the addresses below. You can find more information on the project on the website:

<http://www.ucl.ac.uk/public-health/sphere/spherehome.htm>.

We would appreciate if you could send the completed questionnaire by email to: [claudiac@ensp.unl.pt](mailto:claudiac@ensp.unl.pt) or by fax to FAX number + 351 217582754 by January 29^th^ 2006.

Contacts:

| Dr.ª Claudia Conceição  Tel + 351 217512100; +351 217512160  E-mail: [claudiac@ensp.unl.pt](mailto:claudiac@ensp.unl.pt) | Dr.ª Patricia Barbosa  Tel + 351 217512100; +351 217512156  E-mail: [patbarbosa@ensp.unl.pt](mailto:patbarbosa@ensp.unl.pt) |
| --- | --- |

Address:

Drª. Claudia Conceição

Escola Nacional de Saúde Pública

Avenida Padre Cruz

1600-560 Lisboa

Portugal
